# BioID Reveals Novel Proteins of the *Plasmodium* Parasitophorous Vacuole Membrane

**DOI:** 10.1128/mSphere.00522-17

**Published:** 2018-01-24

**Authors:** Cilly Bernardette Schnider, Damaris Bausch-Fluck, Francis Brühlmann, Volker T. Heussler, Paul-Christian Burda

**Affiliations:** a Institute of Cell Biology, University of Bern, Bern, Switzerland; b Graduate School for Cellular and Biomedical Sciences, University of Bern, Bern, Switzerland; c Department of Biomedical Sciences, Faculty of Medicine & Health Sciences, Macquarie University, Sydney, New South Wales, Australia; d Institute of Molecular Systems Biology, ETH Zurich, Zurich, Switzerland; e Department of Health Sciences and Technology, BMPP, ETH Zurich, Zurich, Switzerland; University at Buffalo

**Keywords:** BioID, blood stage, liver stage, PVM, *Plasmodium*, membranes

## Abstract

During their development within the vertebrate host, *Plasmodium* parasites infect hepatocytes and red blood cells. Within these cells, parasites are surrounded by a parasitophorous vacuole membrane (PVM). The PVM plays an essential role for the interaction of parasites with their host cells; however, only a limited number of proteins of this membrane have been identified so far. This is partially because systematic proteomic analysis of the protein content of the PVM has been difficult in the past, due to difficulties encountered in attempts to separate the PVM from other membranes such as the parasite plasma membrane. In this study, we adapted the BioID technique to *in vitro*-cultivated *Plasmodium berghei* blood stage parasites and utilized the promiscuous biotin ligase BirA* fused to PVM-resident exported protein 1 to biotinylate proteins of the PVM. These we further processed by affinity purification, liquid chromatography-tandem mass spectrometry (LC-MS/MS), and label-free quantitation, leading to a list of 61 known and candidate PVM proteins. Seven proteins were analyzed further during blood and liver stage development. This resulted in the identification of three novel PVM proteins, which were the serine/threonine protein phosphatase UIS2 (PlasmoDB accession no. PBANKA_1328000) and two conserved *Plasmodium* proteins with unknown functions (PBANKA_0519300 and PBANKA_0509000). In conclusion, our report expands the number of known PVM proteins and experimentally validates BioID as a powerful method to screen for novel constituents of specific cellular compartments in *P. berghei*.

**IMPORTANCE** Intracellular pathogens are often surrounded by a host-cell derived membrane. This membrane is modified by the pathogens to their own needs and is crucial for their intracellular lifestyle. In *Plasmodium* parasites, this membrane is referred to as the PVM and only a limited number of its proteins are known so far. Here, we applied in rodent *P. berghei* parasites a method called BioID, which is based on biotinylation of proximal and interacting proteins by the promiscuous biotin ligase BirA*, and demonstrated its usefulness in identification of novel PVM proteins.

## Abstract (precis)

Intracellular pathogens are often surrounded by a host-cell derived membrane. This membrane is modified by the pathogens to their own needs and is crucial for their intracellular lifestyle. In *Plasmodium* parasites, this membrane is referred to as the PVM and only a limited number of its proteins are known so far. Here, we applied in rodent *P. berghei* parasites a method called BioID, which is based on biotinylation of proximal and interacting proteins by the promiscuous biotin ligase BirA*, and demonstrated its usefulness in identification of novel PVM proteins.

## INTRODUCTION

Malaria is a widespread disease putting half of the world’s population at risk of infection and leading to nearly half a million deaths per year (1). The causative agent of the disease is *Plasmodium*, a parasite transmitted by infected *Anopheles* mosquitoes during a blood meal. After injection into the skin of a vertebrate host, *Plasmodium* sporozoites actively enter blood vessels and reach the liver, where they infect hepatocytes. Here they undergo a first round of multiplication. After release from the liver, parasites repeatedly invade and multiply within red blood cells (RBCs), leading to the symptoms of malaria. Within hepatocytes and RBCs, *Plasmodium* parasites reside within a specialized compartment referred to as the parasitophorous vacuole (PV) and are surrounded by the PV membrane (PVM), which is initially formed by invagination of the host cell membrane (HCM) during the process of invasion (reviewed in reference 2). The PVM is extensively remodeled by the parasite, and, as the interface between the parasite and its host cell, it plays fundamental roles in nutrient acquisition, host cell remodeling, waste disposal, environmental sensing, and protection from innate defense mechanisms of the host cell (reviewed in reference 3). At the end of parasite development in hepatocytes and RBCs, parasites induce disruption of the PVM and the HCM, which is essential for their release from host cells and for propagating the infection.

Only a limited number of PVM proteins have been identified so far, and the functions of most of these remain elusive (reviewed in references 3 and 4). These include the earliest known PVM protein, exported protein 1 (EXP1), which contains a classical N-terminal signal peptide and is inserted into the PVM of blood and liver stage parasites with its transmembrane domain, whereby the C terminus faces the host cell cytoplasm (5–8). EXP1 was shown to be refractory to gene deletion, indicating an essential role for asexual blood stage development as gene targeting is performed in this stage (9). In line with its predicted glutathione *S*-transferase activity, EXP1 has the ability to conjugate glutathione onto hematin *in vitro* (10). Furthermore, evidence was provided that the C-terminal portion of EXP1 specifically interacts with host apolipoprotein H and that this interaction is crucial for development of the parasite within the liver (11). Other prominent PVM proteins are the components of the *Plasmodium* translocon of exported proteins (PTEX), mediating the export of proteins into the host cell (12), and proteins belonging to the family of the early transcribed membrane proteins (ETRAMPs) (13).

In general, many more PVM proteins have been identified in blood stage parasites than in liver stage parasites, where only a few PVM proteins are known so far (reviewed in references 3 and 4). Apart from EXP1, these liver stage PVM proteins include two members of the ETRAMP family, UIS3 and UIS4, the knockout of which leads to parasites arrested in early liver stage development (14, 15), or the phospholipase PbPL, whose deletion results in parasites that are impaired in rupturing the liver stage PVM during egress from host hepatocytes (16).

A systematic proteomic analysis of the PVM’s protein composition has been difficult in the past, due to difficulties encountered in attempts to separate the PVM from other membranes such as the parasite plasma membrane (PPM). In this study, we applied the BioID technique to identify novel proteins of the *Plasmodium* PVM. This biochemical approach is based on the promiscuous biotin ligase BirA*, which can be fused to a protein of interest and, upon addition of biotin, leads to the biotinylation of proximal and potentially interacting proteins that can be affinity purified and identified by mass spectrometry (17). Using EXP1 as the bait, we identified 61 known and candidate PVM proteins. We further analyzed a subset of these candidates by endogenous green fluorescent protein (GFP) tagging during blood and liver stage development, leading to the discovery of three novel PVM proteins.

## RESULTS

### Tagging of the PVM-resident EXP1 protein with the BirA* biotin ligase.

**FIG 1:**
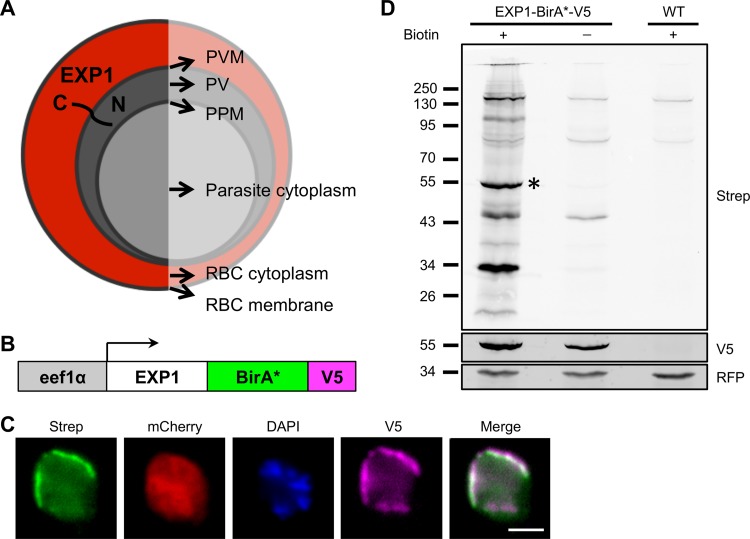
Expression of EXP1-BirA*-V5 leads to biotinylation of proteins at the parasite periphery. (A) Localization of EXP1 within *Plasmodium*-infected erythrocytes. (B) Schematic representation of the expression cassette encoding EXP1 fused to BirA* with a C-terminal V5 tag driven by the constitutive *eef1*α promoter. (C) IFA of EXP1-BirA*-V5 blood stage parasites grown for 18 h in the presence of biotin. Biotinylated proteins were detected by Alexa Fluor 488-labeled streptavidin (Strep; green). The cytosolic mCherry is shown in red and the V5 signal in purple. Parasite nuclei were stained with DAPI and are displayed in blue. Scale bar, 2 μm. (D) Western blot analysis of lysates from EXP1-BirA*-V5 parasites grown for 18 h with or without biotin and WT parasites grown in the presence of biotin. Lysates were probed with fluorescently labeled streptavidin to detect biotinylated proteins. The EXP1-BirA*-V5 fusion protein is predicted to be 55 kDa (asterisk). Cytosolic mCherry detected with an anti-RFP antibody served as a loading control.

To identify novel proteins of the *Plasmodium* PVM, we adapted the BioID approach and fused the biotin ligase BirA* to EXP1, which is a known protein of the PVM in blood and liver stage parasites (Fig. 1A). With this aim, we generated the plasmid pL0017-^C^EXP1-BirA*-V5, encoding an EXP1-BirA* fusion protein with a C-terminal V5 tag under control of the constitutive *P. berghei eef1α* promoter (Fig. 1B). This vector integrates by single-crossover homologous recombination into either the *c-ssu*-rRNA locus or *d-ssu*-rRNA locus of *P. berghei* and conveys resistance to pyrimethamine. We transfected it into blood stage schizonts of marker-free parasites expressing mCherry under the control of the constitutive *P. berghei hsp70* promoter (16). The parental line expressing mCherry was used as a control and is further referred to as the wild type (WT). We obtained transgenic EXP1-BirA*-V5 parasites after drug selection and confirmed successful integration into the genome by PCR (see Fig. S1 in the supplemental material).

10.1128/mSphere.00522-17.1 FIG S1  Integration PCR of EXP1-BirA*-V5 parasites (related to Fig. 1). (A) Schematic representation of the pL0017-based plasmid used to generate EXP1-BirA*-V5 parasites. This vector integrates into the *c*- or the *d-ssu*-rRNA locus via single-crossover recombination and conveys resistance to pyrimethamine. (B) Diagnostic PCR of EXP1-BirA*-V5 parasites. Successful integration of the transfected plasmid into either of two possible loci in the *P. berghei* genome was tested by PCR. Locations of primers used for PCR analysis are shown. For each locus, one primer pair (primer pair 1 and 4 or primer pair 2 and 4, respectively) yields a PCR product of 3 kb if no integration has taken place. In the case of successful integration, the primers are too far apart (>14 kb) to result in a complete PCR product under the chosen conditions. To further confirm integration, additional primer pairs (primer pair 1 and 3 and primer pair 2 and 3) were used, which generated a PCR product of 3 kb only if the plasmid had integrated. All primer sequences are listed in Table S3. Download FIG S1, TIF file, 0.5 MB. Copyright © 2018 Schnider et al. 2018 Schnider et al. This content is distributed under the terms of the Creative Commons Attribution 4.0 International license.

To investigate whether this construct is expressed in blood stages and leads to the biotinylation of PVM proteins, we isolated the blood of mice infected with transgenic EXP1-BirA*-V5 parasites by cardiac puncture and cultivated it in an *in vitro* schizont culture for 18 h in the presence of 150 μM biotin. Biotinylated proteins were visualized by immunofluorescence analysis (IFA) using fluorescently labeled streptavidin. As expected, we observed a strong streptavidin signal, which was peripherally located around parasites and colocalized with the V5-tagged EXP1-BirA* fusion protein (Fig. 1C). We further confirmed this finding by performing Western blot analyses of EXP1-BirA*-V5 parasites grown in the presence or absence of biotin and parental WT parasites cultivated in the presence of biotin. We observed a substantial increase in the levels of biotinylated proteins in lysates of EXP1-BirA*-V5 parasites grown in the presence of biotin in comparison to the WT and no-biotin controls (Fig. 1D), where only very few biotinylated proteins were visible. In conclusion, these data demonstrate that EXP1-BirA*-V5 is active and biotinylates a range of target proteins at the parasite periphery.

### Identification of novel PVM candidate proteins by mass spectrometry.

**FIG 2:**
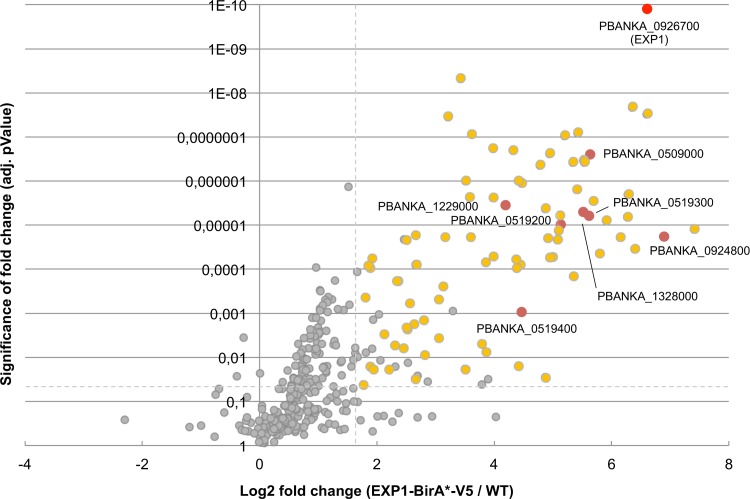
Identification of PVM candidates by quantitative mass spectrometry. A volcano plot depicting quantified proteins is shown. The *x* axis shows the average fold change (log_2_) in protein abundance in EXP1-BirA*-V5 samples in comparison to WT controls, and the *y* axis shows the average adjusted (adj.) *P* value; the data for both were determined on the basis of results from two independent experiments. Proteins meeting the selection criteria (for details, see the main text) and considered significantly enriched in EXP1-BirA*-V5 parasites are displayed in yellow. The bait protein (EXP1; PBANKA_0926700) and candidates selected for further localization studies are highlighted in red. For a complete list of all identified proteins, see Table S1; for a Venn diagram showing the overlap of the data from the two independent experiments, see Fig. S2. Proteins significantly enriched in EXP1-BirA*-V5 parasites and containing a signal peptide and/or at least one transmembrane domain are displayed and further described in Fig. 3 (see also Table S2).

**FIG 3:**
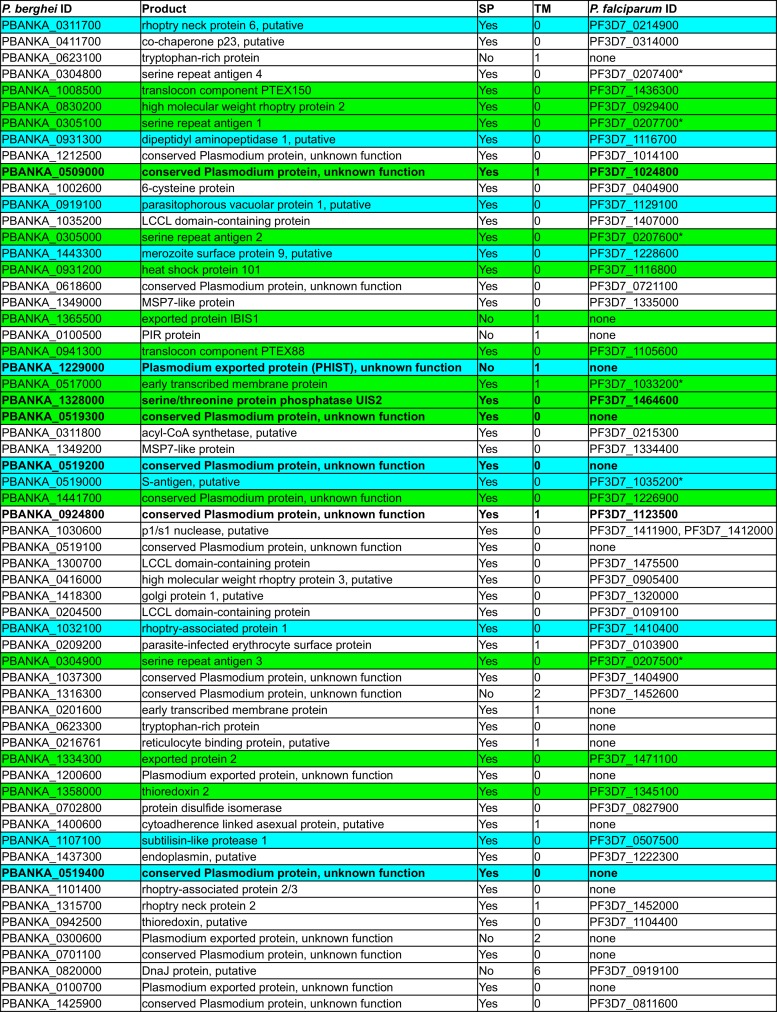
PVM candidates with a signal peptide and/or at least one transmembrane domain. Data corresponding to PVM or PV localization of candidate proteins in *P. berghei* and *P. falciparum* determined on the basis of this work and published studies that used electron and/or fluorescence microscopy techniques for protein localization are indicated. Proteins that showed at least partial PVM localization during blood and/or liver stage development in *P. berghei* or *P. falciparum* are indicated in green, while proteins with at least partial PV localization and no further PVM localization are shown in blue. Candidate proteins analyzed in this study are highlighted in bold. *P. falciparum* orthologs were identified in http://plasmodb.org/plasmo/, and, in some cases, synteny was used as a further indicator to support orthology (indicated by asterisks). Candidates are ordered by increasing average *P* values. For details regarding localizations (including references) see Table S2. ID, identifier; SP, signal peptide. TM, number of transmembrane domains.

To identify the targets labeled by EXP1-BirA*-V5, we performed two independent experiments, in which we subjected biotinylated proteins to affinity purification using streptavidin-conjugated magnetic beads and analyzed them by label-free quantitative mass spectrometry. We compared EXP1-BirA*-V5 parasites to parental WT parasites, both of which were grown in the presence of biotin. Altogether, we identified 1,157 proteins; quantitative information could be extracted for 631 proteins, and 357 were reliably quantified with at least two independent peptide measurements across all samples. Proteins that were at least three times more abundant in EXP1-BirA*-V5 parasites than in WT parasites in both independent experiments, with an adjusted *P* value of below 0.05 as calculated based on analysis of variance (ANOVA) models, were considered significantly enriched in EXP1-BirA*-V5 parasites (Fig. 2; see also Fig. S2 and Table S1 in the supplemental material). Among these 82 proteins, we further excluded all proteins which did not contain a signal peptide and/or at least one transmembrane domain, resulting in a final list of 61 candidate proteins, including several already-identified PVM-resident proteins, such as all components of the PTEX complex (PTEX150, Hsp101, PTEX88, TRX2, and EXP2) and two members of the ETRAMP family (Fig. 3; see also Table S2).

10.1128/mSphere.00522-17.6 TABLE S1  List of all proteins identified by quantitative mass spectrometry in the two independent experiments with a 1% false-discovery rate (related to Fig. 2 and 3). Protein probabilities, sequence coverage, and numbers of unique peptides are indicated for each sample (replicate 1-WT, replicate 1-EXP1-BirA*-V5, replicate 2-WT, and replicate 2-EXP1-BirA*-V5). In addition, the calculated log_2_ fold changes (EXP1-BirA*-V5/WT) and the corresponding adjusted *P* value for the two replicates from which the average log_2_ fold change and the average adjusted *P* values were obtained are indicated. The last column indicates proteins that were significantly enriched in the EXP1-BirA*-V5 sample according to the criteria described in the main text. Download TABLE S1, XLS file, 0.5 MB. Copyright © 2018 Schnider et al. 2018 Schnider et al. This content is distributed under the terms of the Creative Commons Attribution 4.0 International license.

10.1128/mSphere.00522-17.2 FIG S2  Overlap of the two independent experiments (related to Fig. 2). The figure presents a Venn diagram showing the overlap of the two replicates and containing all proteins, which were enriched at least three times in the EXP1-BirA*-V5 parasites in comparison to the WT control parasites, with a *P* value of <0.05. Download FIG S2, TIF file, 0.2 MB. Copyright © 2018 Schnider et al. 2018 Schnider et al. This content is distributed under the terms of the Creative Commons Attribution 4.0 International license.

10.1128/mSphere.00522-17.7 TABLE S2  Localization of PVM candidate proteins in *P. berghei* and *P. falciparum* based on this work and published studies that used electron and/or fluorescence microscopy techniques for protein localization (related to Fig. 3). The localizations given were obtained in asexual blood stage parasites, if not stated otherwise. Proteins that showed at least partial PVM localization during blood and/or liver stage development are indicated in green, while proteins with at least partial PV localization during blood and/or liver stage development and no further PVM localization are shown in blue. Candidate proteins further analyzed in this study are highlighted in bold. *P. falciparum* orthologs were identified in http://plasmodb.org/plasmo/, and, in some cases, synteny was used as a further indicator to support orthology (indicated by asterisks). Candidates are ordered by increasing average *P* values. BS, blood stages; LS, liver stages; GM, gametocytes. Download TABLE S2, PDF file, 0.1 MB. Copyright © 2018 Schnider et al. 2018 Schnider et al. This content is distributed under the terms of the Creative Commons Attribution 4.0 International license.

### Localization of selected candidates by endogenous GFP tagging.

Among the proteins identified by mass spectrometry, we selected seven proteins and determined their localization by C-terminal GFP tagging. We concentrated on candidates that had not yet been localized in *P. berghei* and *P. falciparum* at the time of analysis, most of which were annotated as proteins with unknown function. Each corresponding gene was targeted using the pOB277 vector (18) that integrates by single-crossover homologous recombination into the endogenous locus, leading to expression of a GFP-tagged version of the protein under the control of its endogenous promoter (Fig. S3A). Successful integration into the desired locus was confirmed by PCR (Fig. S3B), and transgenic blood stage parasites were analyzed live by fluorescence microscopy. We focused our analysis on schizonts and free merozoites, as these stages allow discrimination between putative PVM, PV, and PPM localizations. We did not perform further colocalization experiments in the blood stage, as discrimination of PVM, PV, and PPM proteins in this stage by IFA is very difficult to achieve, due to the close proximity of the PVM and the PPM (3). Instead, we decided to include liver stage parasites in our analysis, where such discrimination by IFA is possible. The cytomere stage is particularly suited to localization studies, since parasites are very large at that time point and the PPM has already started to invaginate (19), allowing a clear differentiation between a potential PVM and PV/PPM localization. We therefore concentrated on fully developed cytomere liver stage parasites, which were fixed and stained with an antiserum against EXP1 to perform colocalization analysis by confocal microscopy.

**FIG 4:**
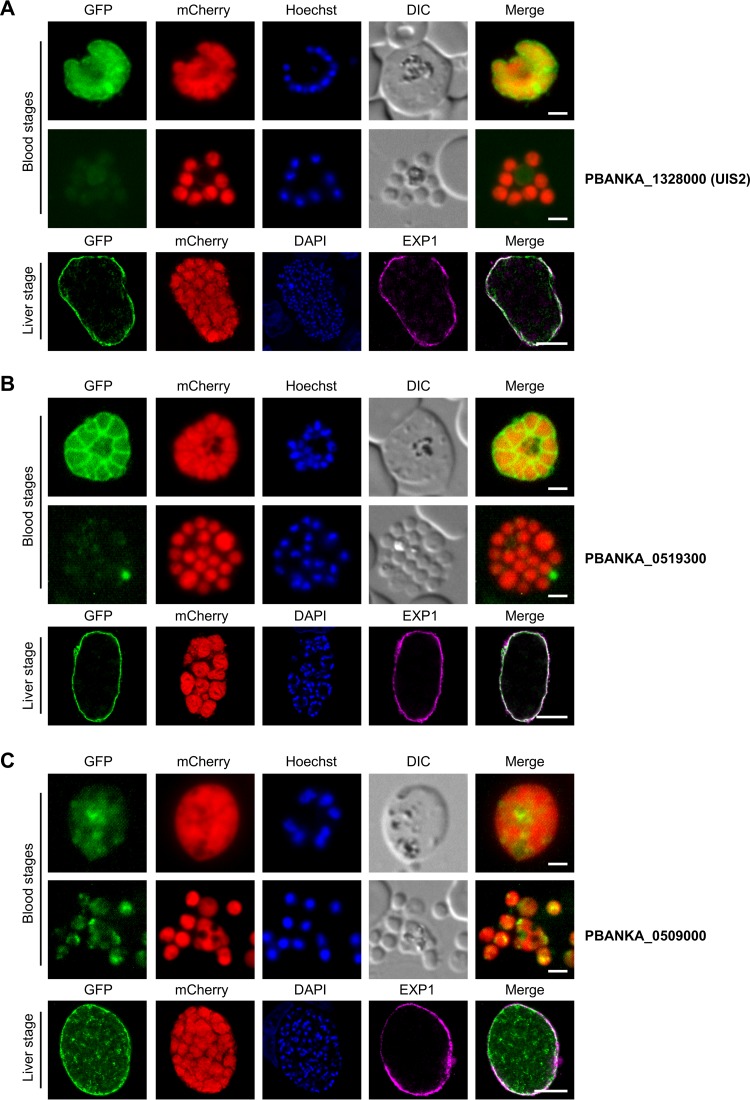
Candidates with a PVM localization. Live-cell microscopy images of developing blood stage merozoites (upper panels) or free blood stage merozoites (middle panels) as well as an IFA of fixed liver stage cytomeres stained with an antiserum against EXP1 (lower panels) are shown for parasites expressing endogenously GFP-tagged PBANKA_1328000 (A), PBANKA_0519300 (B), or PBANKA_0509000 (C). The endogenously GFP-tagged proteins are shown in green, the cytosolic mCherry proteins are shown in red, and the EXP1 signal in the IFA of fixed liver stage parasites is displayed in purple. DNA of Hoechst- or DAPI-stained nuclei is shown in blue. DIC, differential interference contrast. Scale bars for all blood stages; 2 μm; scale bars for the liver stage, 10 μm.

**FIG 5:**
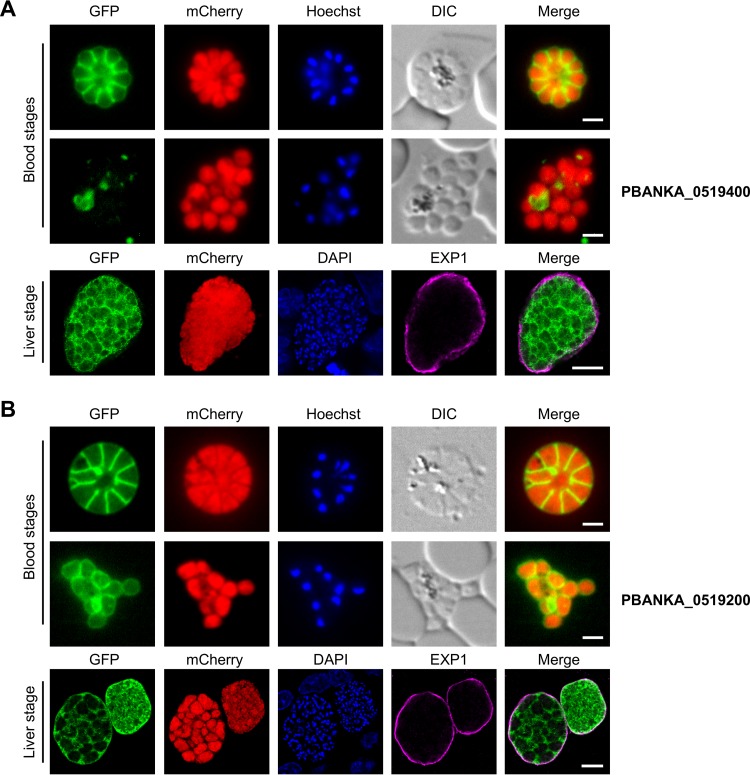
Candidates that are localized to the PV and/or PPM. Live-cell microscopy images of developing blood stage merozoites (upper panels) or free blood stage merozoites (middle panels) as well as an IFA of fixed liver stage cytomeres stained with an antiserum against EXP1 (lower panels) are shown for parasites expressing endogenously GFP-tagged PBANKA_0519400 (A) and PBANKA_0519200 (B). The endogenously GFP-tagged proteins are shown in green, the cytosolic mCherry proteins are shown in red, and the EXP1 signal in the IFA of fixed liver stage parasites is displayed in purple. DNA of Hoechst- or DAPI-stained nuclei is shown in blue. DIC, differential interference contrast. Scale bars for all blood stages, 2 μm; scale bars for the liver stage, 10 μm.

**FIG 6:**
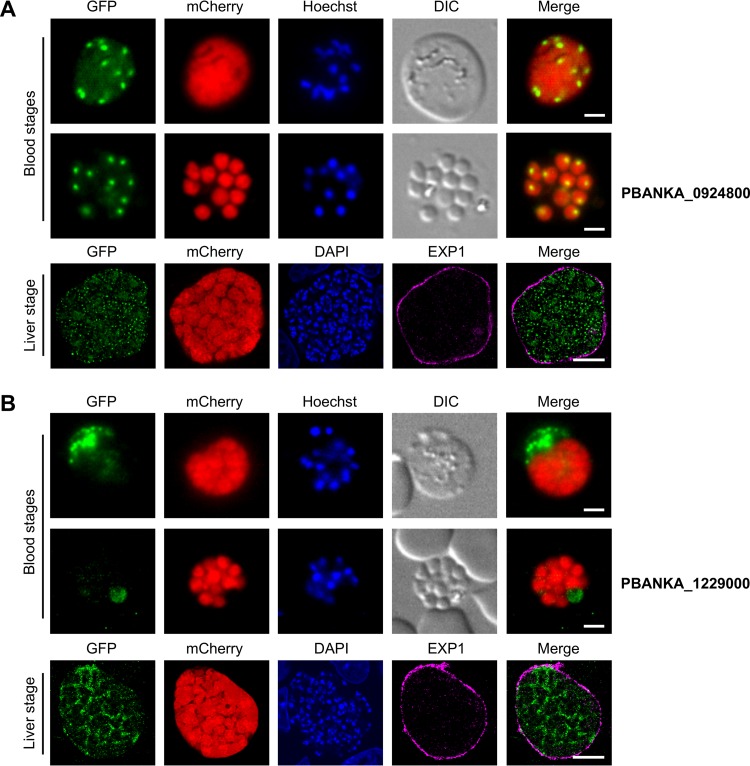
Localization of PBANKA_1229000 and PBANKA_0924800 during blood and liver stage development. Live-cell microscopy images of developing blood stage merozoites (upper panels) or free blood stage merozoites (middle panels) as well as an IFA of fixed liver stage cytomeres stained with an antiserum against EXP1 (lower panels) are shown for parasites expressing endogenously GFP-tagged PBANKA_1229000 (A) and PBANKA_0924800 (B). The endogenously GFP-tagged proteins are shown in green, the cytosolic mCherry proteins are shown in red, and the EXP1 signal in IFAs of fixed liver stage parasites is displayed in purple. DNA of Hoechst- or DAPI-stained nuclei is shown in blue. DIC, differential interference contrast. Scale bars for all blood stages, 2 μm; scale bars for the liver stage, 10 μm.

10.1128/mSphere.00522-17.3 FIG S3  Integration PCR of parasites expressing endogenously GFP-tagged candidate proteins (related to Fig. 4, 5, and 6). (A) Schematic representation of the target locus in WT parasites and in parasites after successful integration (INT) by single-crossover recombination of the pOB277-based vector for endogenous GFP tagging of target genes. Locations of primers used for PCR analysis are shown. For each locus, one primer pair (primer pair 1 and 2) yields a PCR product if no integration has taken place. In the case of successful integration, the primers are too far apart to result in a complete PCR product under the chosen conditions. Another primer pair (primer pair 1 and 3) generates a PCR product only if the plasmid has integrated. (B) Agarose gels with PCR products from genomic DNA of WT controls and the parasite lines indicated above each gel. All primer sequences are listed in Table S3. Download FIG S3, TIF file, 0.8 MB. Copyright © 2018 Schnider et al. 2018 Schnider et al. This content is distributed under the terms of the Creative Commons Attribution 4.0 International license.

### Candidate proteins with PVM localization.

Of the seven GFP-tagged proteins, three proteins were clearly localized to the PVM during liver stage development (Fig. 4). These were the serine/threonine phosphatase UIS2 (PBANKA_1328000) and two conserved *Plasmodium* proteins with unknown function (PBANKA_0519300 and PBANKA_0509000). During blood stage development, UIS2-GFP mainly surrounded developing merozoites as a whole, while free merozoites were not enclosed by a UIS2 signal, suggesting that the peripheral staining in developing schizonts is derived from PVM localization of the protein during blood stage development. In line with this, UIS2 was found to colocalize with EXP1 in liver stage parasites, indicating PVM localization also during this stage of development (Fig. 4A). The GFP signal of PBANKA_0519300 in infected RBCs was found at the parasite periphery and between forming merozoites, pointing to localization of this protein in the PV or PPM and maybe additionally to the PVM. Free merozoites of this parasite line were not surrounded by a GFP signal, excluding PPM localization and suggesting that PBANKA_0519300 is localized to the PV and possibly additionally to the PVM during blood stage development. In liver stage parasites, PBANKA_0519300 localized to the PVM, as confirmed by colocalization with EXP1 (Fig. 4B). PBANKA_0509000 showed weak, hazy staining within the parasite cytoplasm in blood stage parasites; however, it partially colocalized with EXP1 in liver stage cytomeres (Fig. 4C), demonstrating that this protein can be transported to the PVM during parasite development also.

### Candidate proteins with PV/PPM or other localizations.

Two other conserved *Plasmodium* proteins with unknown functions (PBANKA_0519400 and PBANKA_0519200) were found mainly in the PV or PPM during blood and liver stage development (Fig. 5). PBANKA_0519400 surrounded developing blood stage merozoites individually, while no GFP signal was found around free merozoites, pointing to PV localization of this protein during blood stage development. Similarly, in liver stage cytomeres, the protein did not colocalize with EXP1 and instead showed a typical PV/PPM localization (Fig. 5A). To investigate this further, we performed colocalization analysis with the PPM marker protein merozoite surface protein 1 (MSP1). PBANKA_0519400 colocalized with MSP1 only partially and concentrated at the parasite periphery of late liver stage parasites, which is consistent with PV localization also during liver stage development (Fig. S4). The other protein, PBANKA_0519200, also surrounded developing merozoites individually; however, free merozoites of this parasite line were clearly surrounded by a GFP signal, indicating PPM localization of this protein in blood stage parasites. During liver stage development, PBANKA_0519200 did not colocalize with EXP1 and showed typical PV/PPM localization (Fig. 5B). Costaining with PPM marker MSP1 showed partial colocalization with this protein, especially in liver stage merozoites. PBANKA_0519200 also concentrated at the parasite periphery during liver stage development, indicating that this protein localizes additionally to the PV during that stage of development (Fig. S5).

10.1128/mSphere.00522-17.4 FIG S4  IFA of fixed liver stages of PBANKA_0519400 parasites stained with an antiserum against PPM marker MSP1 (related to Fig. 5). The endogenously GFP-tagged protein is shown in green, the cytosolic mCherry in red, and the MSP1 signal in purple. DNA of DAPI-stained nuclei is displayed in blue. Arrows indicate areas where the GFP signal is surrounding PPM marker MSP1, indicating PV localization during liver stage development. Scale bars, 10 μm. Download FIG S4, TIF file, 2.5 MB. Copyright © 2018 Schnider et al. 2018 Schnider et al. This content is distributed under the terms of the Creative Commons Attribution 4.0 International license.

10.1128/mSphere.00522-17.5 FIG S5  IFA of fixed liver stages of PBANKA_0519200 parasites stained with an antiserum against PPM marker MSP1 (related to Fig. 5). The endogenously GFP-tagged protein is shown in green, the cytosolic mCherry in red, and the MSP1 signal in purple. DNA of DAPI-stained nuclei is displayed in blue. Arrows indicate areas where the GFP signal is surrounding PPM marker MSP1, indicating PV localization during liver stage development. Scale bars, 10 μm. Download FIG S5, TIF file, 2 MB. Copyright © 2018 Schnider et al. 2018 Schnider et al. This content is distributed under the terms of the Creative Commons Attribution 4.0 International license.

Another conserved *Plasmodium* protein with unknown function (PBANKA_0924800) showed one single focus per parasite in developing and free blood stage merozoites, suggesting a secretory pathway or invasion organelle location. During liver stage development, the protein also appeared as discrete foci in cytomere stage parasites, indicating localization similar to that observed in blood stage parasites (Fig. 6A). Finally, a *Plasmodium* exported protein (PHIST) with unknown function (PBANKA_1229000) was exported to the host cell cytosol in developing merozoites, while no signal was observed in free merozoites. In the cytomere stage of liver stage parasites, this protein was also secreted by the parasite but mainly localized to the PV (Fig. 6B).

## DISCUSSION

During their development within RBCs and hepatocytes, *Plasmodium* parasites are surrounded by a PVM. This membrane represents the interphase between parasite and host and plays a fundamental role in its interaction with the host cell. In this study, we used BioID in combination with endogenous GFP tagging to identify novel PVM proteins in *P. berghei*.

On the basis of our two BioID experiments, we obtained a list of 61 candidate proteins that were potentially localized to the blood stage PVM. Many of these, such as all components of the PTEX complex, were already known to be localized to this compartment. In general, this confirms the good coverage of our approach. However, a considerable number of the 61 candidate proteins are known not to locate to the PVM, which was evident in the endogenous GFP-tagging approach of this study as well, where four of the seven analyzed proteins were found in other non-PVM compartments such as the PV or the PPM. This occurrence of quite a high number of off-target hits might have derived from the fact that the EXP1-BirA*-V5 fusion protein is already active during its transport through the secretory pathway. During this transport the PPM and the PV have to be crossed and proteins of these compartments as well as other proteins of the secretory pathway might become biotinylated. Furthermore, we currently cannot completely exclude the possibility that the constitutive expression of EXP1 driven by the strong *P. berghei eef1α* promoter or its fusion to the BirA*-V5 protein might interfere with the proper targeting of EXP1 to the PVM. Possibly, this could lead to partial PV localization of the EXP1-BirA* fusion protein, which could biotinylate proteins not only of the PVM but also of the PV and PPM there.

Rather than an in-depth analysis of single candidate proteins, we used endogenous GFP tagging for analysis of seven candidate genes during blood and liver stage development as a proof of concept for our BioID approach. This analysis resulted in the identification of three previously unknown proteins of the liver stage PVM, which were the serine/threonine protein phosphatase UIS2 and two conserved *Plasmodium* proteins with unknown functions (PBANKA_0519300 and PBANKA_0509000).

UIS2 was initially discovered in a screen for genes that display upregulated expression in salivary gland sporozoites (20) and has been shown to be essential for blood stage development in *P. berghei* and *P. falciparum* (21, 22). Similarly to our results obtained in this study, UIS2 also localizes to the PVM of *P. falciparum* blood stage parasites (22). It is interesting that the localization of UIS2 in *P. berghei* blood stages is slightly more diffuse than its localization in *P. falciparum*, which might be due to strain-specific differences in expression. By utilization of a conditional knockout approach, Zhang and colleagues provided evidence that UIS2 plays a role in transformation of *P. berghei* sporozoites to liver stages through a function in dephosphorylating eIF2α-P in the parasite cytoplasm (21). In this regard, our observation that UIS2 localizes to the PVM during blood and liver stage development is very interesting, as it indicates that UIS2 might have another noncytoplasmic and yet-to-be-identified function in PVM biology.

The other two novel proteins of the liver stage PVM, PBANKA_0519300 and PBANKA_0509000, were both shown to be nonessential for blood stage development in *P. berghei* (23, 24), but the function of these two proteins during liver stage development has not been analyzed so far. The *P. falciparum* ortholog of PBANKA_0509000, which was recently named EXP3, was localized to the blood stage PVM as well (22, 24). Remarkably, although likely not essential for *P. falciparum* blood stage development, EXP3 was shown to be part of a newly described exported protein-interacting complex (EPIC), which has been proposed to be involved in the trafficking of virulence determinants to the surface of the infected erythrocyte (24). Thus, the fact that we did localize the *P. berghei* ortholog of EXP3 to the liver stage PVM also is intriguing, as this suggests that an EPIC-like complex could also be involved in trafficking of parasite proteins to the infected hepatocyte, a hypothesis which clearly needs further investigation.

In this study, we used the promiscuous biotin ligase BirA* to identify novel proteins of the *Plasmodium* PVM. A very promising approach for the future might be the application of the engineered enzyme ascorbate peroxidase (APEX). This enzyme can also be fused to a protein of interest and, similarly to BirA*, can lead to biotinylation of nearby proteins, with the big difference of having a labeling time of 1 min, which is much shorter than the many hours of labeling time which are needed to achieve satisfactory biotinylation levels in a BioID experiment (25). The temporal specificity of the biotinylation reaction could thus be increased, and it might become possible to look at the stage-specific proteome of the PVM and to specifically identify proteins involved in PVM remodeling early in infection or to identify PVM-localized proteins involved in membrane disruption processes of late stage parasites during egress from host cells.

The BioID technique has become a powerful and widely used tool in cell biology and has already been applied in studies of several parasite species, including *Trypanosoma brucei* (26), *Toxoplasma gondii* (27–29), and *P. falciparum* (22). It has been used in *P. berghei* studies in an *in vivo* setting, where the biotinylation reaction was carried out in parasite-infected mice (30). Here, we show for the first time the successful application of BioID in *in vitro*-cultivated *P. berghei* blood stage parasites and demonstrate its usefulness in identification of novel proteins of the *Plasmodium* PVM, altogether contributing to our understanding of the molecular makeup of this fascinating interface between a parasite and its host cell.

## MATERIALS AND METHODS

### Ethics statement.

All experiments performed were conducted in strict accordance with the guidelines of the Swiss Tierschutzgesetz (TSchG; Animal Rights Laws) and approved by the ethical committee of the University of Bern (permit number BE109/13).

### Experimental animals.

BALB/c mice used in the experiments were between 6 and 10 weeks of age and were from Harlan Laboratories or Charles River, Inc., or were bred in the central animal facility at the University of Bern. Mosquito feeds were performed on anesthetized mice (Ketavet/Domitor), and all efforts were made to minimize suffering.

### Mosquito infection.

Infection with *P. berghei* parasites was initiated by intraperitoneal injection of blood stabilates. After a parasitemia level of 4% was reached, 40 µl of infected blood (mixed with 160 µl of phosphate-buffered saline [PBS]) was injected intravenously into mice that had been pretreated with an intraperitoneal injection of 200 µl phenylhydrazine (6 mg/ml in PBS) 2 to 4 days prior to infection. At three to four days after infection, mice with a parasitemia level of at least 7% were anesthetized for 1 h to allow feeding of 150 female *Anopheles stephensi* mosquitoes. Mosquitoes were kept at 20.5°C with 80% humidity, and for infection experiments, sporozoites were isolated from infected salivary glands 16 to 27 days post-blood meal.

### Culture and infection of HepG2 cells with *P. berghei.*

HepG2 cells (obtained from the European Cell Culture Collection) were cultured as described previously (31). For infection, 5 × 10^4^ cells were seeded in 24-well plates with coverslips. The next day, *P. berghei* sporozoites were isolated from salivary glands of infected *A. stephensi* mosquitoes and added to HepG2 cells in culture medium additionally containing 2.5 μg/ml amphotericin B (PAA Laboratories). After an incubation period of 2 h, the sporozoite-containing medium was removed and fresh infection medium was added. Subsequently, medium was changed once per day.

### Cloning of DNA constructs.

All PCRs were performed using Phusion DNA polymerase (NEB). PCR products were routinely cloned into pJET1.2 (Fermentas) and confirmed by sequencing (Microsynth). The pL0017-^C^EXP1-BirA*-V5 plasmid was generated by first amplifying the BirA* coding sequence from pcDNA3.1-mycBioID (17) using primers BirA-V5-fw and BirA-V5-rev, whereby a V5 tag was added to the C-terminal end of BirA*. The PCR product was then cloned into pL0017 (32) using BamHI and XbaI restriction sites. Subsequently, the EXP1 coding sequence was amplified from blood stage cDNA using primers EXP1-fw and EXP1-rev and cloned in frame with BirA*-V5 into the pL0017 vector using BamHI and NotI restriction sites. To generate the plasmids for C-terminal endogenous GFP tagging of PVM candidates, the C-terminal homology regions of the candidate genes were amplified by PCR and cloned in frame with GFP into pOB277 (18) using KpnI and ApaI restriction sites. All primer sequences are listed in Table S3 in the supplemental material.

10.1128/mSphere.00522-17.8 TABLE S3  Primers used in this study. Download TABLE S3, XLS file, 0.03 MB. Copyright © 2018 Schnider et al. 2018 Schnider et al. This content is distributed under the terms of the Creative Commons Attribution 4.0 International license.

### Transfection of *P. berghei* parasites and confirmation of correct integration.

The pL0017-^C^EXP1-BirA*-V5 plasmid and the pOB277-based GFP-tagging vectors were linearized using the restriction enzymes listed in Table S4. Linearized plasmids were transfected into blood stage schizonts of *P. berghei* ANKA mCherry_Hsp70_ parasites (16) using standard methods of transfection (33). Transfected parasites were selected by supplementing the drinking water of infected mice with pyrimethamine (Sigma). Parasite genomic DNA was isolated from 0.05% saponin-treated infected red blood cells using a NucleoSpin Blood QuickPure kit (Macherey-Nagel), and successful integration of the plasmid into the genome was confirmed by diagnostic PCR using GoTaq Flexi DNA polymerase (Promega). All primer sequences are listed in Table S3.

10.1128/mSphere.00522-17.9 TABLE S4  Restriction enzymes used for linearization of targeting constructs for parasite transfection. Download TABLE S4, XLS file, 0.03 MB. Copyright © 2018 Schnider et al. 2018 Schnider et al. This content is distributed under the terms of the Creative Commons Attribution 4.0 International license.

### Live-cell imaging and immunofluorescence assay.

Blood stage parasites from schizont cultures or from a drop of tail blood were imaged live, and, for visualization of DNA, 1 µg/ml Hoechst 33342 (Sigma) was added. Blood stage IFA was performed as described previously (19). Primary antibodies were rat anti-red fluorescent protein (anti-RFP) (Chromotek) and mouse anti-V5 (Invitrogen). As secondary antibodies, donkey anti-rat Alexa Fluor 594 (Invitrogen) and goat anti-mouse Cy5 (Dianova) were used. Streptavidin-Alexa Fluor 488 (Invitrogen) and 1 µg/ml DAPI (4′,6-diamidino-2-phenylindole) (Sigma) were applied together with the secondary antibodies, and coverslips were mounted using Dako fluorescent mounting medium (Dako). All blood stage images were taken on a Leica DM5500 B epifluorescence microscope using an HCX Plan-Apochromat 100×/1.4 oil objective, and image processing was performed using ImageJ.

For liver stage IFA, 5 × 10^4^ HepG2 cells were seeded onto coverslips in 24-well plates and infected the following day with *P. berghei* sporozoites. At different time points postinfection, cells were fixed with 4% paraformaldehyde (PFA)–PBS for 20 min at room temperature, followed by permeabilization performed with ice-cold methanol for at least 30 min. Nonspecific binding sites were blocked by incubation in 10% fetal calf serum (FCS)–PBS, followed by incubation with primary antibodies (rabbit anti-GFP [Invitrogen] and chicken anti-EXP1 and rat anti-MSP1 [both generated at the Bernhard Nocht Institute, Hamburg, Germany]) and subsequently with fluorescently labeled secondary antibodies (goat anti-rabbit Alexa Fluor 488 [Invitrogen], goat anti-rat Alexa Fluor 647 [Invitrogen], and donkey anti-chicken Cy5 [Dianova]) diluted in 10% FCS–PBS. DNA was stained with 1 µg/ml DAPI. Labeled cells were mounted on microscope slides with Dako fluorescent mounting medium and analyzed by using a Leica TCS SP8 confocal microscope with an HC PL 100×/1.40 oil objective. Image processing was performed using ImageJ.

### Western blotting.

Western blotting was performed as described previously (34) using rat anti-RFP (Chromotek) and mouse anti-V5 (Invitrogen) as primary antibodies. Goat anti-rat IgG 680LT IRDye and goat anti-mouse IgG 680LT IRDye (both Li-COR) were used as secondary antibodies. Biotinylated proteins were detected using IRDye 800CW-labeled streptavidin (Li-COR).

### BioID of PVM proteins.

Infection with EXP1-BirA*-V5 and WT mCherry_Hsp70_ parasites was initiated by intraperitoneal injection of blood stabilates into two mice per parasite line. After a parasitemia level of 3% to 4% was reached, mice were sacrificed and blood was removed by cardiac puncture and incubated in an overnight schizont culture as previously described (33). Incubation was done for 18 h in the presence or absence of 150 µM biotin (Sigma). Schizonts were enriched on a Nycodenz gradient, and a small sample was taken for Western blotting as described above. Detergent-based extraction and affinity capture of biotinylated proteins were basically done as described before (27); however, incubation on streptavidin Mag Sepharose beads (GE Healthcare) was done at 4°C overnight with continuous agitation.

### Mass spectrometric analysis.

Mass spectrometric analysis was performed by the proteomics and mass spectrometry core facility at the Department of Biomedical Research of the University of Bern. Proteins were reduced, alkylated, and digested with trypsin for 6 h at 37°C on beads. Digests were loaded onto a precolumn (PepMap C_18_) (5-µm pore size; 300 *A*; 300-µm inner diameter [i.d.] by 15-mm length) at a flow rate of 20 µl/min with solvent A (0.1% formic acid–water/acetonitrile [98:2]). After loading, peptides were eluted in back flush mode onto the analytical nanocolumn (C_18_) (5-µm pore size; 300 *A*; 0.075-mm i.d. by 150-mm length) using an acetonitrile gradient of 5% to 40% solvent B (0.1% formic acid–water/acetonitrile [4.9:95]) for 40 min at a flow rate of 400 nl/min. The column effluent was directly coupled to a Fusion Lumos mass spectrometer (Thermo Fischer) via a nanospray electrospray ionization (ESI) source. Data acquisition was made in data-dependent mode with precursor ion scans recorded in a Fourier transform detector (FT) with a resolution of 120,000 (at *m/z* = 400) parallel to top-speed fragment spectra of the most intense precursor ions in the linear trap for a maximum cycle time of 3 s.

### Protein identification and label-free quantitation.

Raw files were converted to mzXML format with ProteoWizard v3.0.6002 (http://proteowizard.sourceforge.net/), searched with comet (v2015.01) (35) against a combined *P. berghei* sequence database consisting of data from http://www.uniprot.org and http://plasmodb.org/plasmo/ (PlasmoDB-28) (with the following parameters: fully tryptic, 2 missed cleavages allowed, 20 ppm precursor mass tolerance, fixed carbidomethylated cysteines, variable modification on methionines), and further processed with the transproteomic pipeline (TPP; v4.7) (36) to obtain identified peptides and a protein list at the false-discovery rate of 1%. Liquid chromatography-tandem mass spectrometry (LC-MS/MS) runs were aligned and precursor intensities were calculated in ProgenesisQI (Nonlinear Dynamics, v2). Protein abundances and significant results of testing EXP1-BirA*-V5 versus WT parasites were calculated with MSstats (v3.2.2) (37) based on ANOVA models for each replicate separately. *P* values were adjusted according to the method of Benjamini and Hochberg. Only proteins with at least two independent peptide measurements were taken into account. Proteins with fold change values of at least 3 versus wild-type results and an adjusted *P* value of below 0.05 in both biological replicates were regarded as significantly enriched in EXP1-BirA*-V5 parasites.

### Data availability.

All data generated or analyzed during this study are included in this published article (and its supplemental material files). The mass spectrometry proteomics data have been deposited into the ProteomeXchange Consortium via the PRIDE partner repository (38) with data set identifier PXD007857.

## References

[1] World Health Organization 2016 World Malaria Report 2016. World Health Organization, Geneva, Switzerland.

[2] De Niz M, Burda PC, Kaiser G, Del Portillo HA, Spielmann T, Frischknecht F, Heussler VT 2017 Progress in imaging methods: insights gained into *Plasmodium* biology. Nat Rev Microbiol 15:37–54. doi:10.1038/nrmicro.2016.158. 27890922

[3] Spielmann T, Montagna GN, Hecht L, Matuschewski K 2012 Molecular make-up of the *Plasmodium* parasitophorous vacuolar membrane. Int J Med Microbiol 302:179–186. doi:10.1016/j.ijmm.2012.07.011. 22898489

[4] Nyboer B, Heiss K, Mueller AK, Ingmundson A 15 9 2017 The *Plasmodium* liver-stage parasitophorous vacuole: a front-line of communication between parasite and host. Int J Med Microbiol doi:10.1016/j.ijmm.2017.09.008. 28964681

[5] Simmons D, Woollett G, Bergin-Cartwright M, Kay D, Scaife J 1987 A malaria protein exported into a new compartment within the host erythrocyte. EMBO J 6:485–491. 2438130 10.1002/j.1460-2075.1987.tb04779.x PMC553420

[6] Ansorge I, Paprotka K, Bhakdi S, Lingelbach K 1997 Permeabilization of the erythrocyte membrane with streptolysin O allows access to the vacuolar membrane of *Plasmodium falciparum* and a molecular analysis of membrane topology. Mol Biochem Parasitol 84:259–261. doi:10.1016/S0166-6851(96)02806-X. 9084046

[7] Sanchez GI, Rogers WO, Mellouk S, Hoffman SL 1994 *Plasmodium falciparum*: exported protein-1, a blood stage antigen, is expressed in liver stage parasites. Exp Parasitol 79:59–62. doi:10.1006/expr.1994.1060. 8050527

[8] Charoenvit Y, Mellouk S, Sedegah M, Toyoshima T, Leef MF, De la Vega P, Beaudoin RL, Aikawa M, Fallarme V, Hoffman SL 1995 *Plasmodium* *yoelii*: 17-kDa hepatic and erythrocytic stage protein is the target of an inhibitory monoclonal antibody. Exp Parasitol 80:419–429. doi:10.1006/expr.1995.1054. 7729477

[9] Maier AG, Rug M, O’Neill MT, Brown M, Chakravorty S, Szestak T, Chesson J, Wu Y, Hughes K, Coppel RL, Newbold C, Beeson JG, Craig A, Crabb BS, Cowman AF 2008 Exported proteins required for virulence and rigidity of *Plasmodium falciparum*-infected human erythrocytes. Cell 134:48–61. doi:10.1016/j.cell.2008.04.051. 18614010 PMC2568870

[10] Lisewski AM, Quiros JP, Ng CL, Adikesavan AK, Miura K, Putluri N, Eastman RT, Scanfeld D, Regenbogen SJ, Altenhofen L, Llinás M, Sreekumar A, Long C, Fidock DA, Lichtarge O 2014 Supergenomic network compression and the discovery of EXP1 as a glutathione transferase inhibited by artesunate. Cell 158:916–928. doi:10.1016/j.cell.2014.07.011. 25126794 PMC4167585

[11] Sá E, Cunha C, Nyboer B, Heiss K, Sanches-Vaz M, Fontinha D, Wiedtke E, Grimm D, Przyborski JM, Mota MM, Prudêncio M, Mueller A-K 2017 *Plasmodium berghei* EXP-1 interacts with host apolipoprotein H during *Plasmodium* liver-stage development. Proc Natl Acad Sci U S A 114:E1138–E1147. 10.1073/pnas.1606419114 PMC5320984 28137845

[12] de Koning-Ward TF, Gilson PR, Boddey JA, Rug M, Smith BJ, Papenfuss AT, Sanders PR, Lundie RJ, Maier AG, Cowman AF, Crabb BS 2009 A newly discovered protein export machine in malaria parasites. Nature 459:945–949. doi:10.1038/nature08104. 19536257 PMC2725363

[13] Spielmann T, Fergusen DJP, Beck HP 2003 etramps, a new *Plasmodium falciparum* gene family coding for developmentally regulated and highly charged membrane proteins located at the parasite-host cell interface. Mol Biol Cell 14:1529–1544. doi:10.1091/mbc.E02-04-0240. 12686607 PMC153120

[14] Mueller AK, Labaied M, Kappe SHI, Matuschewski K 2005 Genetically modified *Plasmodium* parasites as a protective experimental malaria vaccine. Nature 433:164–167. doi:10.1038/nature03188. 15580261

[15] Mueller AK, Camargo N, Kaiser K, Andorfer C, Frevert U, Matuschewski K, Kappe SHI 2005 *Plasmodium* liver stage developmental arrest by depletion of a protein at the parasite-host interface. Proc Natl Acad Sci U S A 102:3022–3027. doi:10.1073/pnas.0408442102. 15699336 PMC548321

[16] Burda PC, Roelli MA, Schaffner M, Khan SM, Janse CJ, Heussler VT 2015 A *Plasmodium* phospholipase is involved in disruption of the liver stage parasitophorous vacuole membrane. PLoS Pathog 11:e1004760. doi:10.1371/journal.ppat.1004760. 25786000 PMC4364735

[17] Roux KJ, Kim DI, Raida M, Burke B 2012 A promiscuous biotin ligase fusion protein identifies proximal and interacting proteins in mammalian cells. J Cell Biol 196:801–810. doi:10.1083/jcb.201112098. 22412018 PMC3308701

[18] Patzewitz EM, Guttery DS, Poulin B, Ramakrishnan C, Ferguson DJP, Wall RJ, Brady D, Holder AA, Szöőr B, Tewari R 2013 An ancient protein phosphatase, SHLP1, is critical to microneme development in *Plasmodium* ookinetes and parasite transmission. Cell Rep 3:622–629. doi:10.1016/j.celrep.2013.01.032. 23434509 PMC3617505

[19] Burda PC, Schaffner M, Kaiser G, Roques M, Zuber B, Heussler VT 2017 A *Plasmodium* plasma membrane reporter reveals membrane dynamics by live-cell microscopy. Sci Rep 7:9740. doi:10.1038/s41598-017-09569-4. 28851956 PMC5575152

[20] Matuschewski K, Ross J, Brown SM, Kaiser K, Nussenzweig V, Kappe SHI 2002 Infectivity-associated changes in the transcriptional repertoire of the malaria parasite sporozoite stage. J Biol Chem 277:41948–41953. doi:10.1074/jbc.M207315200. 12177071

[21] Zhang M, Mishra S, Sakthivel R, Fontoura BMA, Nussenzweig V 2016 UIS2: a unique phosphatase required for the development of *Plasmodium* liver stages. PLoS Pathog 12:e1005370. doi:10.1371/journal.ppat.1005370. 26735921 PMC4712141

[22] Khosh-Naucke M, Becker J, Mesén-Ramírez P, Kiani P, Birnbaum J, Fröhlke U, Jonscher E, Schlüter H, Spielmann T 2017 Identification of novel parasitophorous vacuole proteins in *P. falciparum* parasites using BioID. Int J Med Microbiol. doi:10.1016/j.ijmm.2017.07.007. 28784333

[23] Fonager J, Pasini EM, Braks JAM, Klop O, Ramesar J, Remarque EJ, Vroegrijk IOCM, van Duinen SG, Thomas AW, Khan SM, Mann M, Kocken CHM, Janse CJ, Franke-Fayard BMD 2012 Reduced CD36-dependent tissue sequestration of *Plasmodium*-infected erythrocytes is detrimental to malaria parasite growth *in vivo*. J Exp Med 209:93–107. doi:10.1084/jem.20110762. 22184632 PMC3260870

[24] Batinovic S, McHugh E, Chisholm SA, Matthews K, Liu B, Dumont L, Charnaud SC, Schneider MP, Gilson PR, de Koning-Ward TF, Dixon MWA, Tilley L 2017 An exported protein-interacting complex involved in the trafficking of virulence determinants in *Plasmodium*-infected erythrocytes. Nat Commun 8:16044. doi:10.1038/ncomms16044. 28691708 PMC5508133

[25] Rhee H-W, Zou P, Udeshi ND, Martell JD, Mootha VK, Carr SA, Ting AY 2013 Proteomic mapping of mitochondria in living cells via spatially restricted enzymatic tagging. Science 339:1328–1331. 23371551 10.1126/science.1230593 PMC3916822

[26] Morriswood B, Havlicek K, Demmel L, Yavuz S, Sealey-Cardona M, Vidilaseris K, Anrather D, Kostan J, Djinovic-Carugo K, Roux KJ, Warren G 2013 Novel bilobe components in *Trypanosoma brucei* identified using proximity-dependent biotinylation. Eukaryot Cell 12:356–367. doi:10.1128/EC.00326-12. 23264645 PMC3571296

[27] Chen AL, Kim EW, Toh JY, Vashisht AA, Rashoff AQ, Van C, Huang AS, Moon AS, Bell HN, Bentolila LA, Wohlschlegel JA, Bradley PJ 2015 Novel components of the *Toxoplasma* inner membrane complex revealed by BioID. mBio 6:e02357-14. doi:10.1128/mBio.02357-14. 25691595 PMC4337574

[28] Gaji RY, Johnson DE, Treeck M, Wang M, Hudmon A, Arrizabalaga G 2015 Phosphorylation of a myosine motor by TgCDPK3 facilitates rapid initiation of motility during *Toxoplasma gondii* egress. PLoS Pathog 11:e1005268. doi:10.1371/journal.ppat.1005268. 26544049 PMC4636360

[29] Nadipuram SM, Kim EW, Vashisht AA, Lin AH, Bell HN, Coppens I, Wohlschlegel JA, Bradley PJ 2016 *In vivo* biotinylation of the *Toxoplasma* parasitophorous vacuole reveals novel dense granule proteins important for parasite growth and pathogenesis. mBio 7:e00808-16. doi:10.1128/mBio.00808-16. 27486190 PMC4981711

[30] Kehrer J, Frischknecht F, Mair GR 2016 Proteomic analysis of the *Plasmodium berghei* Gametocyte Egressome and Vesicular bioID of osmiophilic body proteins identifies merozoite TRAP-like protein (MTRAP) as an essential factor for parasite transmission. Mol Cell Proteomics 15:2852–2862. doi:10.1074/mcp.M116.058263. 27371728 PMC5013303

[31] Graewe S, Rankin KE, Lehmann C, Deschermeier C, Hecht L, Froehlke U, Stanway RR, Heussler V 2011 Hostile takeover by *Plasmodium*: reorganization of parasite and host cell membranes during liver stage egress. PLoS Pathog 7:e1002224. doi:10.1371/journal.ppat.1002224. 21909271 PMC3164640

[32] Franke-Fayard B, Trueman H, Ramesar J, Mendoza J, Van Der Keur M, Van Der Linden R, Sinden RE, Waters AP, Janse CJ 2004 A *Plasmodium berghei* reference line that constitutively expresses GFP at a high level throughout the complete life cycle. Mol Biochem Parasitol 137:23–33. doi:10.1016/j.molbiopara.2004.04.007. 15279948

[33] Janse CJ, Ramesar J, Waters AP 2006 High efficiency transfection and drug selection of genetically transformed blood stages of the rodent malaria parasite *Plasmodium berghei*. Nat Protoc 1:346–356. doi:10.1038/nprot.2006.53. 17406255

[34] Wacker R, Eickel N, Schmuckli-Maurer J, Annoura T, Niklaus L, Khan SM, Guan J-L, Heussler VT 2017 LC3-association with the parasitophorous vacuole membrane of *Plasmodium berghei* liver stages follows a noncanonical autophagy pathway. Cell Microbiol 19:e12754. doi:10.1111/cmi.12754. 28573684

[35] Eng JK, Jahan TA, Hoopmann MR 2013 Comet: an open-source MS/MS sequence database search tool. Proteomics 13:22–24. doi:10.1002/pmic.201200439. 23148064

[36] Deutsch EW, Mendoza L, Shteynberg D, Farrah T, Lam H, Tasman N, Sun Z, Nilsson E, Pratt B, Prazen B, Eng JK, Martin DB, Nesvizhskii AI, Aebersold R 2010 A guided tour of the trans-proteomic pipeline. Proteomics 10:1150–1159. doi:10.1002/pmic.200900375. 20101611 PMC3017125

[37] Choi M, Chang CY, Clough T, Broudy D, Killeen T, MacLean B, Vitek O 2014 MSstats: an R package for statistical analysis of quantitative mass spectrometry-based proteomic experiments. Bioinformatics 30:2524–2526. doi:10.1093/bioinformatics/btu305. 24794931

[38] Vizcaíno JA, Csordas A, del-Toro N, Dianes JA, Griss J, Lavidas I, Mayer G, Perez-Riverol Y, Reisinger F, Ternent T, Xu Q-W, Wang R, Hermjakob H 2016 2016 update of the PRIDE database and its related tools. Nucleic Acids Res 44:D447–D456. 26527722 10.1093/nar/gkv1145 PMC4702828

