# Supplementary figures and images for: BioID Reveals Novel Proteins of the Plasmodium Parasitophorous Vacuole Membrane
Source: mSphere. 2018 Jan 24;3(1):e00522-17. doi: 10.1128/mSphere.00522-17 (PMC5784244; doi:10.1128/mSphere.00522-17)

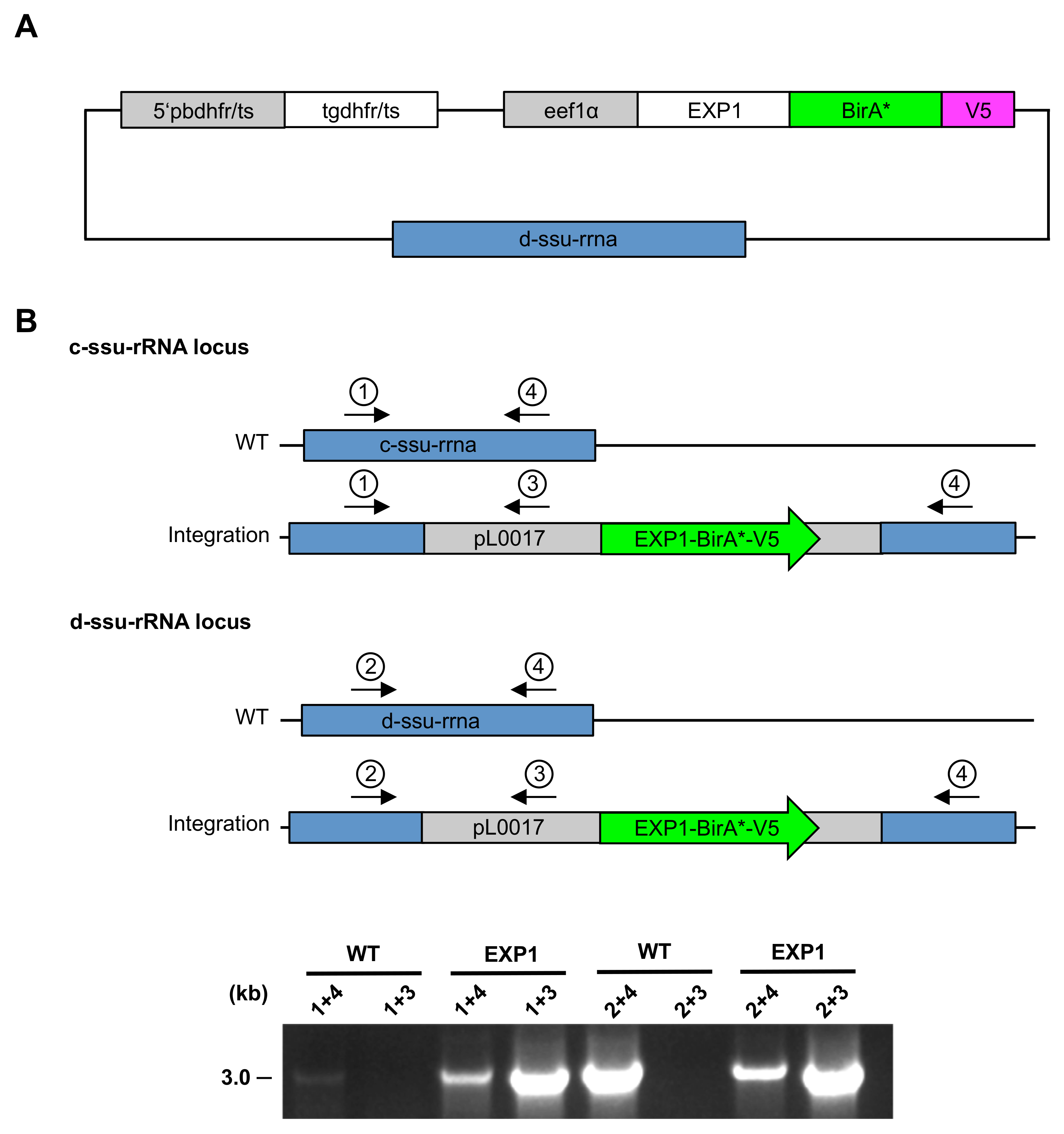

Supplement: FIG S1 [file sph001182463sf1.tif]

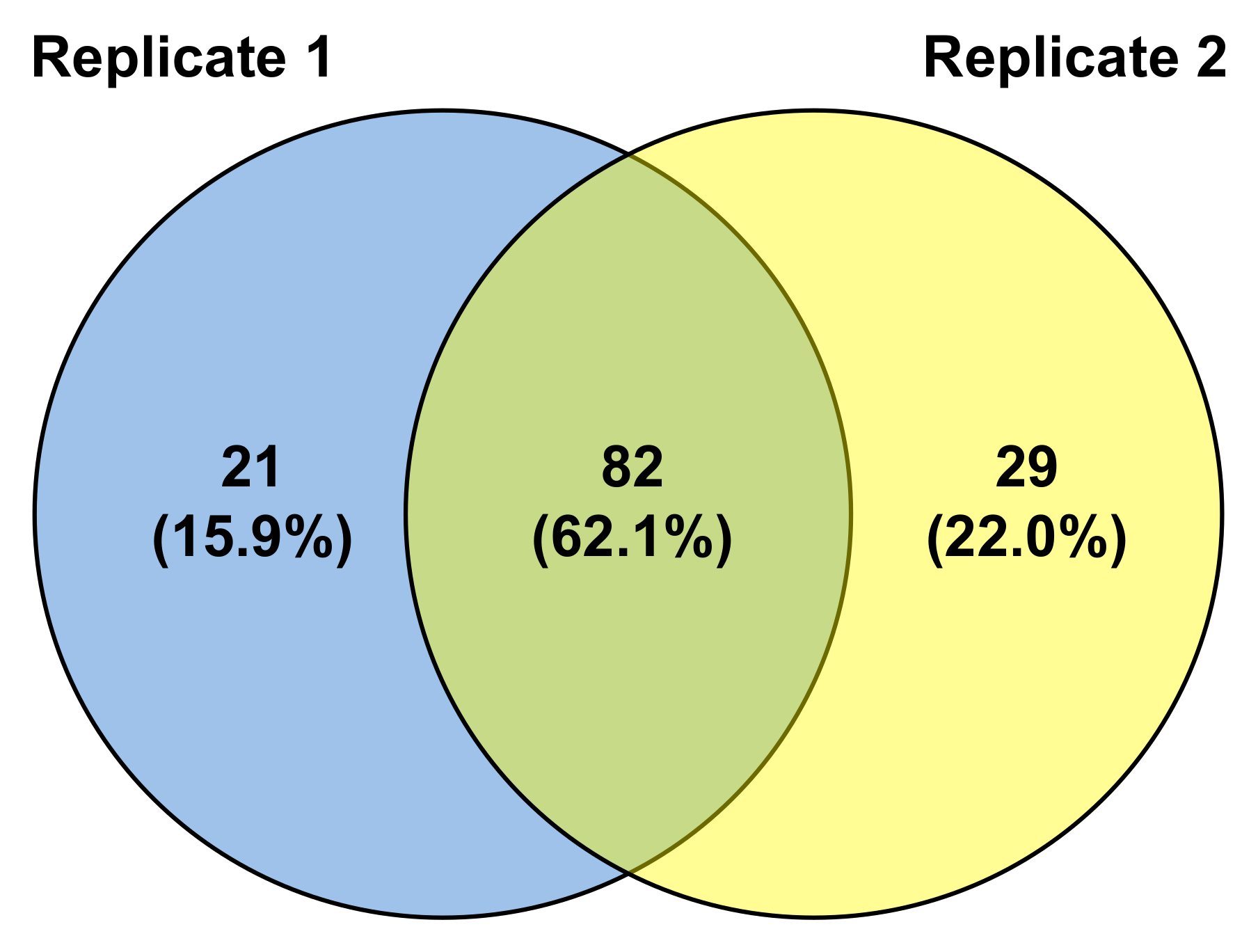

Supplement: FIG S2 [file sph001182463sf2.tif]

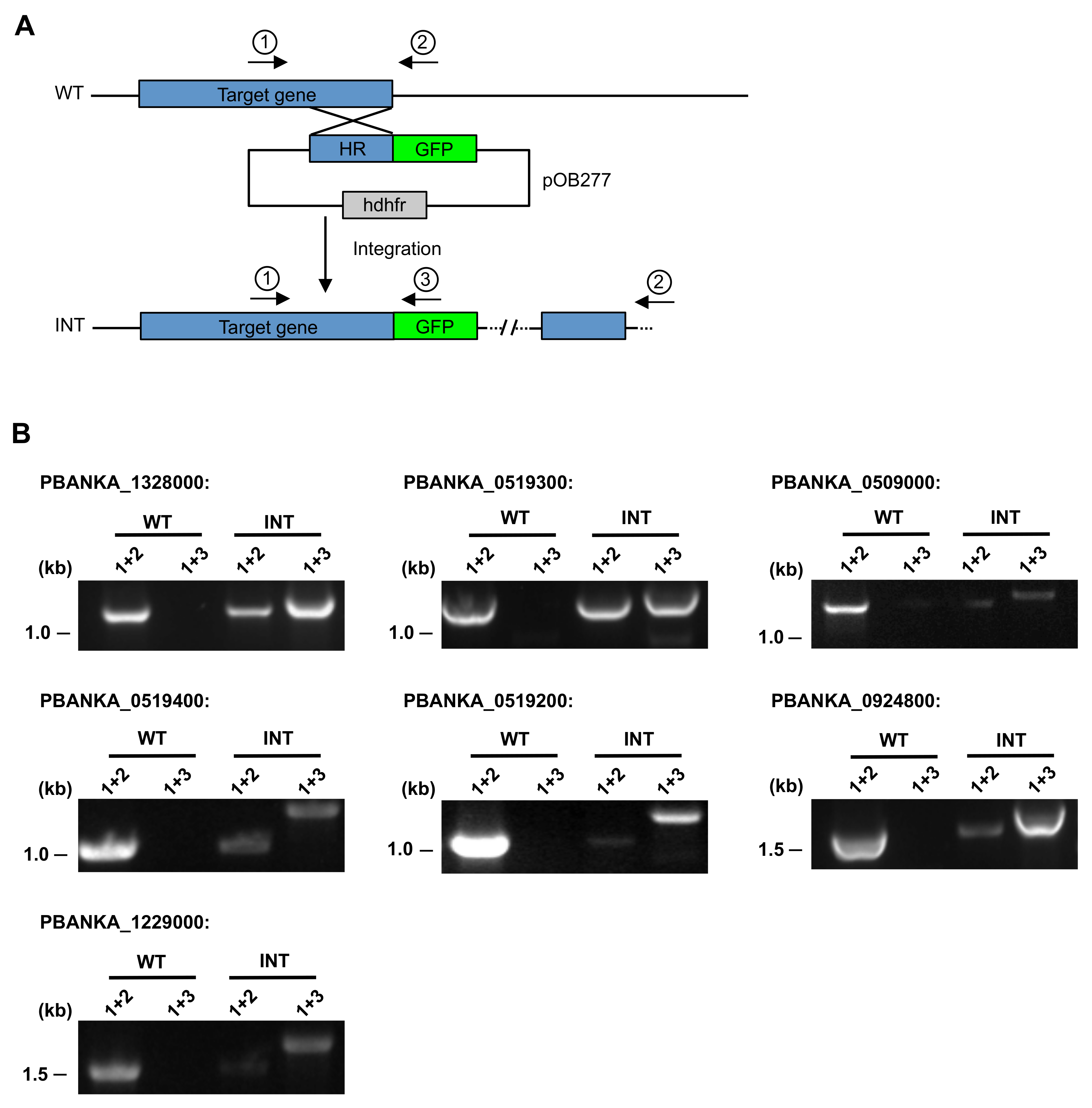

Supplement: FIG S3 [file sph001182463sf3.tif]

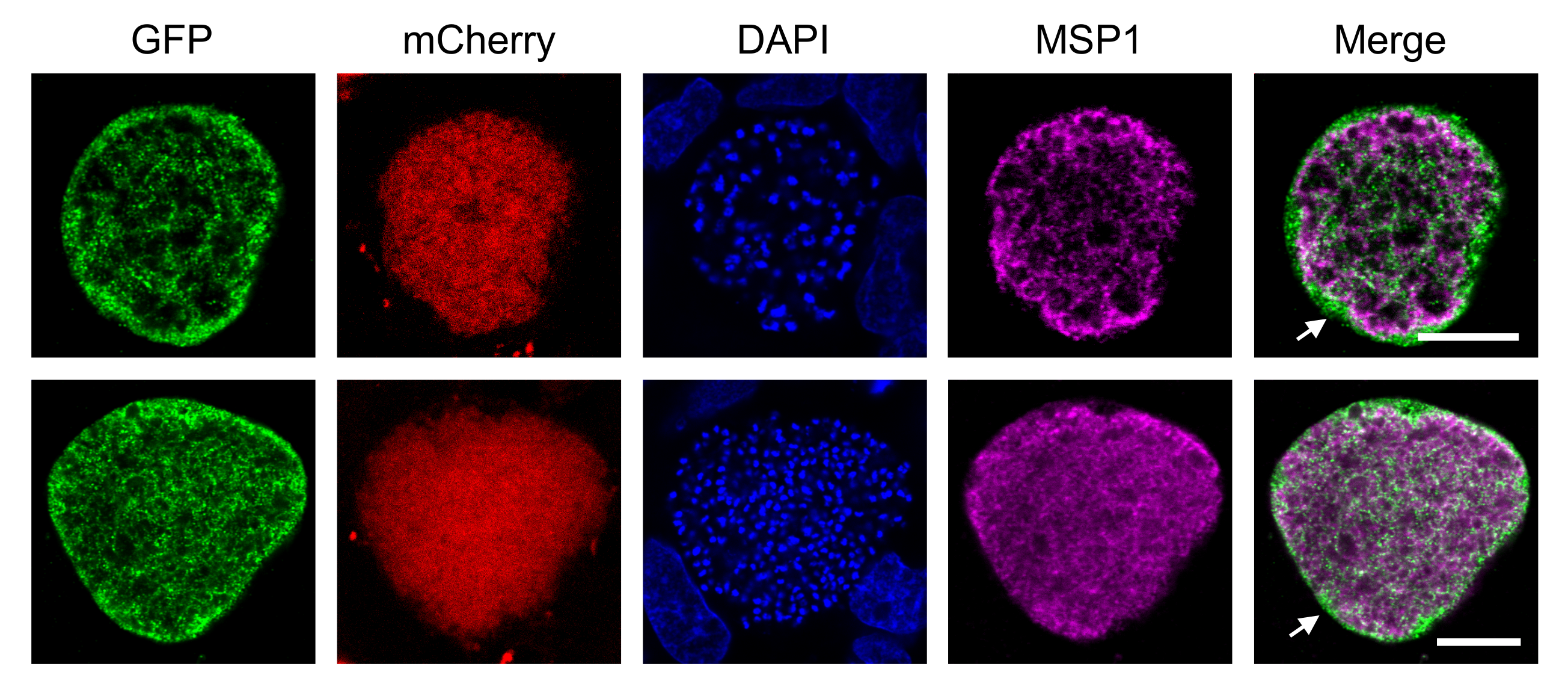

Supplement: FIG S4 [file sph001182463sf4.tif]

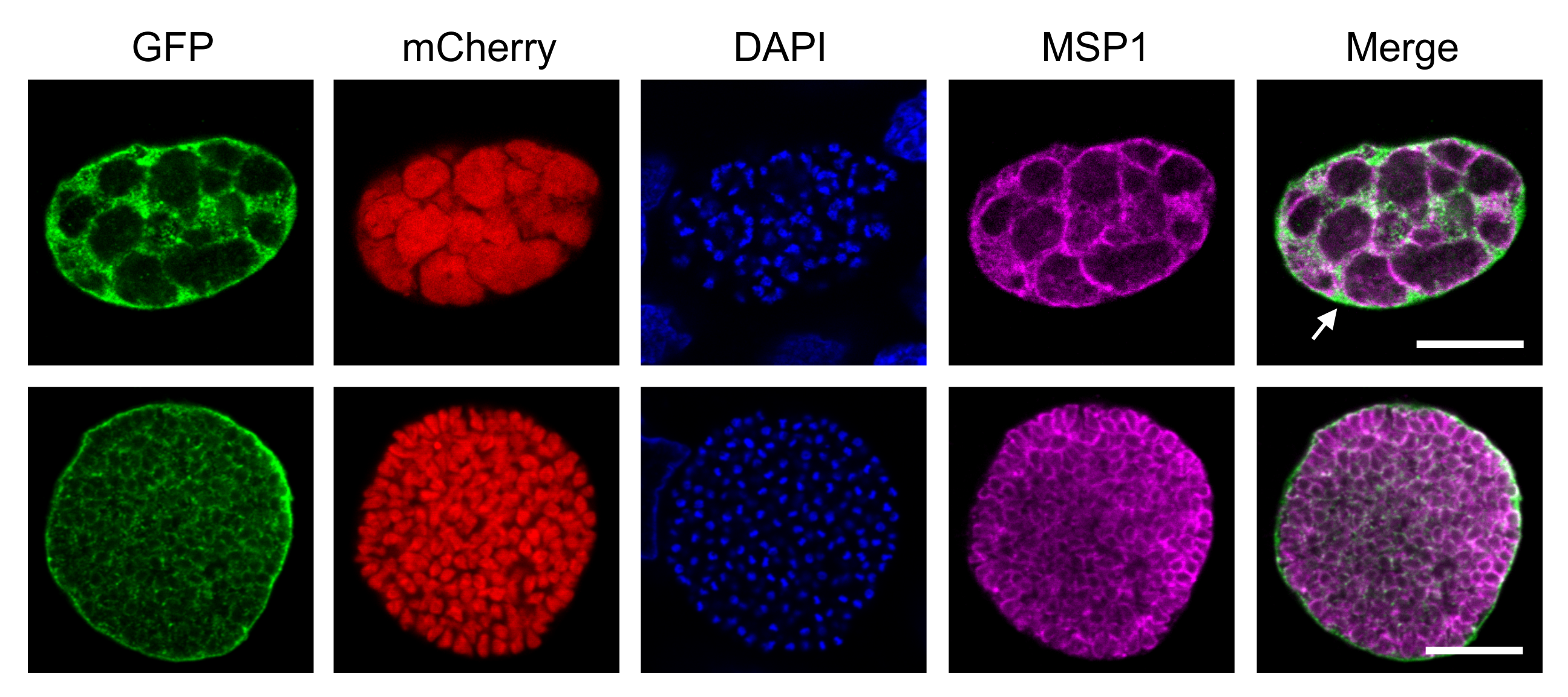

Supplement: FIG S5 [file sph001182463sf5.tif]
