# Supplementary material for: BioID Reveals Novel Proteins of the Plasmodium Parasitophorous Vacuole Membrane
Source: mSphere. 2018 Jan 24;3(1):e00522-17. doi: 10.1128/mSphere.00522-17 (PMC5784244; doi:10.1128/mSphere.00522-17)
Supplement: TABLE S2 [file sph001182463st2.pdf]

**Table S2: Localization of PVM candidate proteins in *P. berghei* and *P. falciparum* based on this work and published studies that used electron and/or fluorescence microscopy techniques for protein localization.**

| ***P. berghei* ID** | **Product** | ***P. falciparum* ID** | **Localization(s) with Reference(s)** |
|---|---|---|---|
| PBANKA_0311700 | rhoptry neck protein 6, putative | PF3D7_0214900 | Pf: rhoptry neck, PV (1) |
| PBANKA_0411700 | co-chaperone p23, putative | PF3D7_0314000 | Pf: cytoplasmic (2) |
| PBANKA_0623100 | tryptophan-rich protein | none | Pb: exported (3) |
| PBANKA_0304800 | serine repeat antigen 4 | PF3D7_0207400* | |
| PBANKA_1008500 | translocon component PTEX150 | PF3D7_1436300 | Pb: PV/PVM (4), Pf: PVM (5) |
| PBANKA_0830200 | high molecular weight rhoptry protein 2 | PF3D7_0929400 | Pf: rhoptries/PVM (6) |
| PBANKA_0305100 | serine repeat antigen 1 | PF3D7_0207700* | Pb: PVM in LS (7), Pf: PV (8) |
| PBANKA_0931300 | dipeptidyl aminopeptidase 1, putative | PF3D7_1116700 | Pf: food vacuole/PV (9) |
| PBANKA_1212500 | conserved Plasmodium protein, unknown function | PF3D7_1014100 | |
| **PBANKA_0509000** | **conserved Plasmodium protein, unknown function** | **PF3D7_1024800** | **Pb: diffuse cytoplasmic in BS, PVM in LS (this study), Pf: PVM (10, 11)** |
| PBANKA_1002600 | 6-cysteine protein | PF3D7_0404900 | Pf: merozoite surface (12) |
| PBANKA_0919100 | parasitophorous vacuolar protein 1, putative | PF3D7_1129100 | Pf: PV (10) |
| PBANKA_1035200 | LCCL domain-containing protein | PF3D7_1407000 | Pb: cytoplasmic in GM (13), Pf: PPM of GM (14) |
| PBANKA_0305000 | serine repeat antigen 2 | PF3D7_0207600* | Pb: PVM/internal structures in LS (7), Pf: PV (15) |
| PBANKA_1443300 | merozoite surface protein 9, putative | PF3D7_1228600 | Pf: merozoite surface, PV (16) |
| PBANKA_0931200 | heat shock protein 101 | PF3D7_1116800 | Pb: PV/PVM (4), Pf: PVM (5) |
| PBANKA_0618600 | conserved Plasmodium protein, unknown function | PF3D7_0721100 | |
| PBANKA_1349000 | MSP7-like protein | PF3D7_1335000 | Pf: trophozoite surface (17) |
| PBANKA_1365500 | exported protein IBIS1 | none | Pb: PVM in LS, membranous structures in RBC cytoplasm (18) |
| PBANKA_0100500 | PIR protein | none | |
| PBANKA_0941300 | translocon component PTEX88 | PF3D7_1105600 | Pb: PV/PVM, Pf: PVM (4) |
| **PBANKA_1229000** | **Plasmodium exported protein (PHIST), unknown function** | **None** | **Pb: exported in BS, PV in LS (18, this study)** |
| PBANKA_0517000 | early transcribed membrane protein | PF3D7_1033200* | Pf: PVM (19) |
| **PBANKA_1328000** | **serine/threonine protein phosphatase UIS2** | **PF3D7_1464600** | **Pb: PVM in BS and LS (this study), Pf: PVM (11)** |
| **PBANKA_0519300** | **conserved Plasmodium protein, unknown function** | **none** | **Pb: PV/PVM in BS, PVM in LS (this study)** |

| | | | |
|---|---|---|---|
| PBANKA_0311800 | acyl-CoA synthetase, putative | PF3D7_0215300 | |
| PBANKA_1349200 | MSP7-like protein | PF3D7_1334400 | Pf: trophozoite surface (17) |
| **PBANKA_0519200** | **conserved Plasmodium protein, unknown function** | **none** | **Pb: PPM/PV in BS and LS (this study)** |
| PBANKA_0519000 | S-antigen, putative | PF3D7_1035200* | Pf: PV (20) |
| PBANKA_1441700 | conserved Plasmodium protein, unknown function | PF3D7_1226900 | Pf: PV, PVM (10, 11) |
| **PBANKA_0924800** | **conserved Plasmodium protein, unknown function** | **PF3D7_1123500** | **Pb: one focus per parasite in BS and LS (this study), Pf: peripheral/foci within parasite (11)** |
| PBANKA_1030600 | p1/s1 nuclease, putative | PF3D7_1411900, PF3D7_1412000 | |
| PBANKA_0519100 | conserved Plasmodium protein, unknown function | none | |
| PBANKA_1300700 | LCCL domain-containing protein | PF3D7_1475500 | Pb: punctate cytoplasmic pattern in GM (21), Pf: PPM in GM (14) |
| PBANKA_0416000 | high molecular weight rhoptry protein 3, putative | PF3D7_0905400 | Pf: rhoptries (22) |
| PBANKA_1418300 | golgi protein 1, putative | PF3D7_1320000 | Pb: rhoptries (23), Pf: Golgi apparatus (24) |
| PBANKA_0204500 | LCCL domain-containing protein | PF3D7_0109100 | Pb: punctate cytoplasmic pattern in GM (21), poles of GM (25) |
| PBANKA_1032100 | rhoptry-associated protein 1 | PF3D7_1410400 | Pf: rhoptries/PV (26) |
| PBANKA_0209200 | parasite-infected erythrocyte surface protein | PF3D7_0103900 | |
| PBANKA_0304900 | serine repeat antigen 3 | PF3D7_0207500* | Pb: PVM/cytoplasmic in LS (27), Pf: PV/PVM (28) |
| PBANKA_1037300 | conserved Plasmodium protein, unknown function | PF3D7_1404900 | Pb: cytoplasmic (13) |
| PBANKA_1316300 | conserved Plasmodium protein, unknown function | PF3D7_1452600 | |
| PBANKA_0201600 | early transcribed membrane protein | none | |
| PBANKA_0623300 | tryptophan-rich protein | none | |
| PBANKA_0216761 | reticulocyte binding protein, putative | none | |
| PBANKA_1334300 | exported protein 2 | PF3D7_1471100 | Pb: PV/PVM (4), Pf: PVM (29) |
| PBANKA_1200600 | Plasmodium exported protein, unknown function | none | Pb: cytoplasmic (3) |
| PBANKA_1358000 | thioredoxin 2 | PF3D7_1345100 | Pb: partial PV/PVM and internal structures (4) |
| PBANKA_0702800 | protein disulfide isomerase | PF3D7_0827900 | |
| PBANKA_1400600 | cytoadherence linked asexual protein, putative | none | Pb: rhoptries (3) |
| PBANKA_1107100 | subtilisin-like protease 1 | PF3D7_0507500 | Pb: (30, 31) punctate pattern in BS and LS, Pf: exonemes/PV (32) |
| PBANKA_1437300 | endoplasmin, putative | PF3D7_1222300 | Pf: ER (33) |
| **PBANKA_0519400** | **conserved Plasmodium protein, unknown function** | **none** | **Pb: PV in BS and LS (this study)** |

| | | | |
|---|---|---|---|
| PBANKA_1101400 | rhoptry-associated protein 2/3 | none | Pb: rhoptries (23) |
| PBANKA_1315700 | rhoptry neck protein 2 | PF3D7_1452000 | Pb: rhoptries (23), Pf: rhoptries (34) |
| PBANKA_0942500 | thioredoxin, putative | PF3D7_1104400 | |
| PBANKA_0300600 | Plasmodium exported protein, unknown function | none | Pb: vesicular localization in GM (13) |
| PBANKA_0701100 | conserved Plasmodium protein, unknown function | none | |
| PBANKA_0820000 | DnaJ protein, putative | PF3D7_0919100 | |
| PBANKA_0100700 | Plasmodium exported protein, unknown function | none | |
| PBANKA_1425900 | conserved Plasmodium protein, unknown function | PF3D7_0811600 | |

**Supplemental References:**


1. Proellocks NI, Kats LM, Sheffield DA, Hanssen E, Black CG, Waller KL, Coppel RL. 2009. Characterisation of PfRON6, a *Plasmodium falciparum* rhoptry neck protein with a novel cysteine-rich domain. Int J Parasitol 39:683–692.
2. Wiser MF. 2003. A *Plasmodium* homologue of cochaperone p23 and its differential expression during the replicative cycle of the malaria parasite. Parasitol Res 90:166–70.
3. Pasini EM, Braks JA, Fonager J, Klop O, Aime E, Spaccapelo R, Otto TD, Berriman M, Hiss JA, Thomas AW, Mann M, Janse CJ, Kocken CHM, Franke-Fayard B. 2013. Proteomic and genetic analyses demonstrate that *Plasmodium berghei* blood stages export a large and diverse repertoire of proteins. Mol Cell Proteomics 12:426–48.
4. Matthews K, Kalanon M, Chisholm SA, Sturm A, Goodman CD, Dixon MWA, Sanders PR, Nebl T, Fraser F, Haase S, McFadden GI, Gilson PR, Crabb BS, de Koning-Ward TF. 2013. The *Plasmodium* translocon of exported proteins (PTEX) component thioredoxin-2 is important for maintaining normal blood-stage growth. Mol Microbiol 89:1167–86.
5. de Koning-Ward TF, Gilson PR, Boddey JA, Rug M, Smith BJ, Papenfuss AT, Sanders PR, Lundie RJ, Maier AG, Cowman AF, Crabb BS. 2009. A newly discovered protein export machine in malaria parasites. Nature 459:945–9.
6. Hiller NL, Akompong T, Morrow JS, Holder AA, Haldar K. 2003. Identification of a stomatin orthologue in vacuoles induced in human erythrocytes by malaria parasites. A role for microbial raft proteins in apicomplexan vacuole biogenesis. J Biol Chem 278:48413–21.
7. Putrianti ED, Schmidt-Christensen A, Arnold I, Heussler VT, Matuschewski K, Silvie O. 2010. The *Plasmodium* serine-type SERA proteases display distinct expression patterns and non-essential in vivo roles during life cycle progression of the malaria parasite. Cell Microbiol 12:725–739.
8. Miller SK, Good RT, Drew DR, Delorenzi M, Sanders PR, Hodder AN, Speed TP, Cowman AF, de Koning-Ward TF, Crabb BS. 2002. A subset of *Plasmodium* falciparum SERA genes are expressed and appear to play an important role in the erythrocytic cycle. J Biol Chem 277:47524–32.
9. Klemba M, Gluzman I, Goldberg DE. 2004. A *Plasmodium falciparum* dipeptidyl aminopeptidase I participates in vacuolar hemoglobin degradation. J Biol Chem 279:43000–7.
10. Batinovic S, McHugh E, Chisholm SA, Matthews K, Liu B, Dumont L, Charnaud SC, Schneider MP, Gilson PR, de Koning-Ward TF, Dixon MWA, Tilley L. 2017. An exported protein-interacting complex involved in the trafficking of virulence determinants in *Plasmodium*-infected erythrocytes. Nat Commun 8:16044.
11. Khosh-Naucke M, Becker J, Mesén-Ramírez P, Kiani P, Birnbaum J, Fröhlke U, Jonscher E, Schlüter H, Spielmann T. 2017. Identification of novel parasitophorous vacuole proteins in *P. falciparum* parasites using BioID. Int J Med Microbiol.
12. Taechalertpaisarn T, Crosnier C, Bartholdson SJ, Hodder AN, Thompson J, Bustamante LY, Wilson DW, Sanders PR, Wright GJ, Rayner JC, Cowman AF, Gilson PR, Crabb BS. 2012. Biochemical and functional analysis of two *Plasmodium falciparum*

blood-stage 6-cys proteins: P12 and P41. PLoS One 7:e41937.
13. Kehrer J, Frischknecht F, Mair GR. 2016. Proteomic Analysis of the *Plasmodium berghei* Gametocyte Egressome and Vesicular bioID of Osmiophilic Body Proteins Identifies Merozoite TRAP-like Protein (MTRAP) as an Essential Factor for Parasite Transmission. Mol Cell Proteomics 15:2852–2862.
14. Pradel G, Hayton K, Aravind L, Iyer LM, Abrahamsen MS, Bonawitz A, Mejia C, Templeton TJ. 2004. A multidomain adhesion protein family expressed in *Plasmodium falciparum* is essential for transmission to the mosquito. J Exp Med 199:1533–44.
15. Delplace P, Fortier B, Tronchin G, Dubremetz JF, Vernes A. 1987. Localization, biosynthesis, processing and isolation of a major 126 kDa antigen of the parasitophorous vacuole of *Plasmodium falciparum*. Mol Biochem Parasitol 23:193–201.
16. Chulay JD, Lyon JA, Haynes JD, Meierovics AI, Atkinson CT, Aikawa M. 1987. Monoclonal antibody characterization of *Plasmodium falciparum* antigens in immune complexes formed when schizonts rupture in the presence of immune serum. J Immunol 139:2768–74.
17. Mello K, Daly TM, Morrisey J, Vaidya AB, Long CA, Bergman LW. 2002. A multigene family that interacts with the amino terminus of *Plasmodium* MSP-1 identified using the yeast two-hybrid system. Eukaryot Cell 1:915–25.
18. Ingmundson A, Nahar C, Brinkmann V, Lehmann MJ, Matuschewski K. 2012. The exported *Plasmodium berghei* protein IBIS1 delineates membranous structures in infected red blood cells. Mol Microbiol 83:1229–43.
19. Spielmann T, Fergusen DJP, Beck H-P. 2003. etramps, a new *Plasmodium falciparum* gene family coding for developmentally regulated and highly charged membrane proteins located at the parasite-host cell interface. Mol Biol Cell 14:1529–44.
20. Culvenor JG, Crewther PE. S-antigen localization in the erythrocytic stages of *Plasmodium falciparum*. J Protozool 37:59–65.
21. Saeed S, Carter V, Tremp AZ, Dessens JT. 2010. *Plasmodium berghei* crystalloids contain multiple LCCL proteins. Mol Biochem Parasitol 170:49–53.
22. Sherling ES, Knuepfer E, Brzostowski JA, Miller LH, Blackman MJ, Ooij C van. 2017. The *Plasmodium falciparum* rhoptry protein RhopH3 plays essential roles in host cell invasion and nutrient uptake. Elife 6.
23. Tufet-Bayona M, Janse CJ, Khan SM, Waters AP, Sinden RE, Franke-Fayard B. 2009. Localisation and timing of expression of putative *Plasmodium berghei* rhoptry proteins in merozoites and sporozoites. Mol Biochem Parasitol 166:22–31.
24. Hallée S, Richard D. 2015. Evidence that the Malaria Parasite *Plasmodium falciparum* Putative Rhoptry Protein 2 Localizes to the Golgi Apparatus throughout the Erythrocytic Cycle. PLoS One 10:e0138626.
25. Scholz SM, Simon N, Lavazec C, Dude M-A, Templeton TJ, Pradel G. 2008. PfCCp proteins of *Plasmodium falciparum*: gametocyte-specific expression and role in complement-mediated inhibition of exflagellation. Int J Parasitol 38:327–40.
26. Richard D, Kats LM, Langer C, Black CG, Mitri K, Boddey JA, Cowman AF, Coppel RL. 2009. Identification of rhoptry trafficking determinants and evidence for a novel sorting mechanism in the malaria parasite *Plasmodium falciparum*. PLoS Pathog 5:e1000328.
27. Schmidt-Christensen A, Sturm A, Horstmann S, Heussler VT. 2008. Expression and processing of *Plasmodium berghei* SERA3

during liver stages. Cell Microbiol 10:1723–1734.
28. Ruecker A, Shea M, Hackett F, Suarez C, Hirst EMA, Milutinovic K, Withers-Martinez C, Blackman MJ. 2012. Proteolytic activation of the essential parasitophorous vacuole cysteine protease SERA6 accompanies malaria parasite egress from its host erythrocyte. J Biol Chem 287:37949–63.
29. Johnson D, Günther K, Ansorge I, Benting J, Kent A, Bannister L, Ridley R, Lingelbach K. 1994. Characterization of membrane proteins exported from *Plasmodium falciparum* into the host erythrocyte. Parasitology 109 ( Pt 1):1–9.
30. Suarez C, Volkmann K, Gomes AR, Billker O, Blackman MJ. 2013. The Malarial Serine Protease SUB1 Plays an Essential Role in Parasite Liver Stage Development. PLoS Pathog 9:1–16.
31. Tawk L, Lacroix C, Gueirard P, Kent R, Gorgette O, Thiberge S, Mercereau-Puijalon O, Ménard R, Barale JC. 2013. A key role for *Plasmodium* subtilisin-like SUB1 protease in egress of malaria parasites from host hepatocytes. J Biol Chem 288:33336–33346.
32. Yeoh S, O'Donnell RA, Koussis K, Dluzewski AR, Ansell KH, Osborne SA, Hackett F, Withers-Martinez C, Mitchell GH, Bannister LH, Bryans JS, Kettleborough CA, Blackman MJ. 2007. Subcellular Discharge of a Serine Protease Mediates Release of Invasive Malaria Parasites from Host Erythrocytes. Cell 131:1072–1083.
33. Yam XY, Birago C, Fratini F, Di Girolamo F, Raggi C, Sargiacomo M, Bachi A, Berry L, Fall G, Currà C, Pizzi E, Breton CB, Ponzi M. 2013. Proteomic analysis of detergent-resistant membrane microdomains in trophozoite blood stage of the human malaria parasite *Plasmodium falciparum*. Mol Cell Proteomics 12:3948–61.
34. Cao J, Kaneko O, Thongkukiatkul A, Tachibana M, Otsuki H, Gao Q, Tsuboi T, Torii M. 2009. Rhoptry neck protein RON2 forms a complex with microneme protein AMA1 in *Plasmodium falciparum* merozoites. Parasitol Int 58:29–35.
